# “Burnout felt inevitable”: Experiences of university staff in educating the nursing and allied health workforce during the first COVID-19 waves

**DOI:** 10.3389/fpubh.2023.1082325

**Published:** 2023-01-30

**Authors:** Lisa O'Brien, Josephine Tighe, Nastaran Doroud, Sarah Barradell, Leah Dowling, Adrian Pranata, Charlotte Ganderton, Robin Lovell, Roger Hughes

**Affiliations:** Department of Nursing and Allied Health, School of Health Sciences, Swinburne University of Technology, Hawthorn, VIC, Australia

**Keywords:** education, workforce, nursing, allied health, pandemic, COVID-19

## Abstract

**Introduction:**

Maintaining progress in the face of looming burnout during the first 2 years of the COVID-19 pandemic was crucial for the health workforce, including those educating the next generation of health professionals. The experiences of students and healthcare practitioners have been explored to a greater degree than the experiences of university-based health professional educators.

**Methods:**

This qualitative study examined the experiences of nursing and allied health academics at an Australian University during COVID-19 disruptions in 2020 and 2021 and describes the strategies that academics and/or teams implemented to ensure course continuity. Academic staff from nursing, occupational therapy, physiotherapy, and dietetics courses at Swinburne University of Technology, Australia provided narratives regarding the key challenges and opportunities they faced.

**Results:**

The narratives highlighted the strategies generated and tested by participants amidst rapidly changing health orders and five common themes were identified: disruption; stress; stepping up, strategy and unexpected positives, lessons, and legacy impacts. Participants noted challenges related to student engagement in online learning and ensuring the acquisition of discipline-specific practical skills during periods of lock-down. Staff across all disciplines reported increased workload associated with converting teaching to on-line delivery, sourcing alternative fieldwork arrangements, and dealing with high levels of student distress. Many reflected on their own expertise in using digital tools in teaching and their beliefs about the effectiveness of distance teaching for health professional training. Ensuring students were able to complete required fieldwork hours was particularly challenging due to constantly changing public health orders and conditions and staffing shortages at health services. This was in addition to illness and isolation requirements further impacting the availability of teaching associates for specialist skills classes.

**Discussion:**

Solutions such as remote and blended learning telehealth, and simulated placements were rapidly implemented in some courses especially where fieldwork could not be rescheduled or amended at the health settings. The implications and recommendations for educating and ensuring competence development in the health workforce during times when usual teaching methods are disrupted are discussed.

## Introduction and context

The COVID-19 pandemic had a profound effect on Australia's education and healthcare sectors (1, 2). Melbourne (Victoria) faced one of the longest and most arduous series of lockdowns (3), altering the delivery of traditional face to face teaching and learning activities in nursing and allied health tertiary education for 257 days across 6 separate lock-down periods between March 30th 2020 and October 26th 2021. During this period, government and university directives required teaching and learning activities to be modified in a range of ways to ensure compliance with public health orders. This resulted in 171 days of entirely online learning with infrequent exceptions for activities such as “super-workshops” conducted with full Personal Protective Equipment (PPE) and a further 82 days where only practical classes were permitted face-to-face with strict safety measures. Consequently, for almost two full academic calendar years, nursing and allied health students were denied or had restricted access to the university facilities (e.g., laboratories, libraries) and educators were required to transition most teaching to a fully online or blended format. The loss of fieldwork practice had the potential for significant bottlenecks for nursing and allied health student course progression, and timely graduation and entry to the health workforce (4). Maintaining a steady flow of qualified health professionals into the health workforce helps meet the health needs of communities addressing existing workforce shortages, fatigue and burnout reported across health professions (1, 5).

Blended approaches to teaching and learning (e.g., online lectures with practical in-class teaching), have been common in health professional education in the previous decade (6). The COVID-19 interruptions, however, required virtual delivery of all teaching, including practical skill-based content, simulations, and fieldwork placements. This was challenging, as these traditionally have involved face-to-face teaching and assessment (7). Complicating matters further, a significant proportion of clinical placements in nursing and allied health were canceled or postponed in 2020-21 in response to government mandates. The reasons for canceling placements prior to the availability of vaccines included density limits (number of people per square meter of clinical space), insufficient amount of PPE available for students, concerns of COVID-19 transmission (health directions meant students could not look after COVID patients or work on COVID wards) and redeployment of clinicians to COVID-19 related work (8–10). In response, the Australian government collaborated with the Australian Health Practitioner Regulation Agency (AHPRA) to publish guidance to allow the return of health profession students to clinical placements and selected on-campus practical learning and teaching activities in mid 2020, albeit with safety measures in place including contact tracing, strict density limits, restricted flow of personnel within learning spaces, appropriate PPE, social distancing, and deep cleaning (11).

Much of the literature published to date focuses on the impact on students of the sudden switch to COVID-safe teaching arrangements, which were generally of limited duration (6, 12–20) or focused on specific responses to eLearning (21, 22) with medicine most represented. Very little of the literature has been empirical in nature (17). In allied health, Peart et al. (18) explored the experiences of Australian occupational therapy educators who supervised students on placement, whereas Plummer et al. (19) and Tajane (20) investigated the experiences of transnational and Indian physiotherapy faculty respectively. All three studies highlight challenges related to expectations, the limitations of the online learning medium and needing to adapt and pivot in real time while simultaneously master instructional technologies. This study adds an interdisciplinary perspective and aims to explore the experiences of nursing and allied health academics at a Melbourne university that encountered significant and sustained impediments to usual course delivery methods over 2 years.

## Materials and methods

### Methodology

This qualitative study explored the experiences of academic staff during the disruption, to describe the strategies tested, and to generate recommendations for ensuring competence development in the health workforce. The COREQ (Consolidated Criteria for Reporting Qualitative Research) Guidelines (23) informed the reporting of this study.

### Participants

Academic staff (*n* = 20) from the Department of Nursing and Allied Health at Swinburne University of Technology (SUT) were invited to participate using purposive sampling. People were eligible to participate if they were teaching or coordinating units with health professional programs at SUT or other Victorian universities during 2020 and 2021. We aimed to recruit at least one academic representative from each of the health professional courses at SUT (nursing, occupational therapy, physiotherapy, and dietetics) to identify commonalities and differences in the experiences of challenges, opportunities and strategies. Potentially eligible participants were contacted *via* email and provided with an explanatory statement regarding the research and an invitation to participate. No incentives or benefits were offered for participation. Consent was implied by participants responding to an anonymous Qualtrics survey link to answer the study questions. Participants were informed prior to data collection about how information would be analyzed and were assured that their comments would be anonymous.

### Data collection

The survey was administered across September 2022 using a structured format (questions are presented in Table 1). Participants were asked to provide written narrative responses to 10 open-ended questions, which enabled participants to individually reflect on their experiences over the previous 2 years. These were submitted anonymously *via* Qualtrics, and participants could choose to answer some or all questions. It was anticipated that completion of responses would take approximately 30–45 min but some people reported taking more than 2 h.

**Table 1:** Narrative guide.

1. What were the key challenges faced during 2020–21?
2. What were the opportunities that arose during this time?
3. What were the strategies/solutions generated and tested in your department? a. Which of these were effective? 1. Are there some that you will keep doing? b. What didn't work, and why?
4. How would you describe student engagement in online learning?
5. How did you ensure the acquisition of discipline-specific practical skills during periods of lock-down?
6. What were the impacts (if any) on your workload? E.g., converting teaching to on-line delivery, sourcing alternative fieldwork arrangements, and dealing with high levels of student distress.
7. How confident/competent were you in using digital tools for teaching prior to this? How did this change over the 2 years?
8. How effective do you think distance teaching is for health professional training for: b. Learning course content? c. Developing competence to practice?
9. What solutions did you try for ensuring students were able to complete required fieldwork hours? a. What were the main challenges to implementing these? b. What worked?
10. What recommendations do you have for educating and ensuring competence development in the health workforce during times when usual teaching methods are disrupted?

### Data management and analysis

Two researchers (LO'B and RH) independently conducted initial manual analyses of the written narratives using a systematic process for line-by-line coding data in which statements were analyzed, compared for similarities and differences, and categorized into clusters of meaning that represented a phenomenon of interest (24). We then compared final themes and categories to review thematic and conceptual consistency. Initial coding was broadly consistent, and subthemes and themes were generated and refined during discussions. Any disagreements were resolved by consensus moderation between the two researchers.

The movement from transcript to codes, subthemes and themes is illustrated in Table 2. To enhance trustworthiness of the data, participants were given an opportunity to provide further comment following the identification of themes and subthemes by researchers. Comments were received about one of the themes (Stepping up) and these were considered in the final account of the theme.

**Table 2:** Example of movement from transcript to theme.

**Transcript excerpt**	**Codes**	**Subtheme**	**Theme**
As an example, a tutorial that usually takes 3.5–4 h needed 4.5–5 h in an online mode. Another huge impact on staff workload was the additional feedback for practical classes, placement support and sourcing out alternative placement options in the event of cancellation. For instance, there normally was 1 h per students for placement support; during the public health restrictions, this almost doubled with [the] need to provide ongoing support for students on placement, placement agencies and problem-solve for alternative solutions when a placement was put on hold.	Lack of recognition–“hidden” workload	Workload uplift	Stepping up
		Innovating and reimagining	
	Need to rewrite /restructure content for online delivery		
	Constant problem solving		

### Ethical considerations

This study was approved by the Human Research and Ethics Committee at Swinburne University of Technology (Ref 20226687-10879).

## Results

Seven participants representing teaching staff (including some with fieldwork coordination responsibilities) from two-year entry-to-practice Masters programs (three from physiotherapy, two from occupational therapy, and one from dietetics) and one from the three-year Bachelor of Nursing provided written narratives. We have chosen not to provide demographic details or attribute quotes to protect the anonymity of the participants; however, the findings reflect the sample as a whole and at least one quote has been chosen from each participant's narrative.

**Figure 1:**
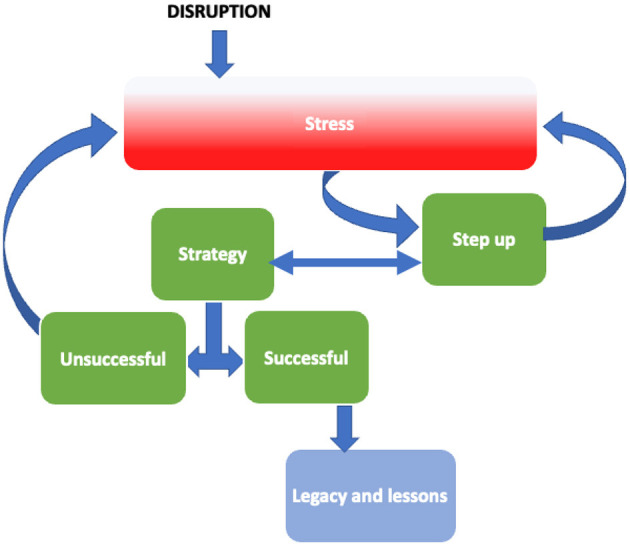
Major themes from narratives.

There were five interlinked major themes (each with multiple sub-themes) that captured the experience of delivering curricula and arranging clinical fieldwork in an environment of constant and unpredictable change. These themes were: Disruption, Stress, Stepping up, Strategy, and Legacy impacts/lessons. Table 3 summarizes the major themes and sub-themes. Many themes and sub-themes appeared to be causative, most obviously the disruption causing additional workload, uncertainty and isolation leading to feelings of stress, fatigue, and burnout. This thematic interconnectivity is illustrated in Figure 1. The themes are described below and illustrated with key quotes.

**Table 3:** Major themes and sub-themes.

**Theme**	**Subthemes**
Disruption	Abrupt and forced change
	Constant change
	Uncertainty
Stress	Lack of preparedness
	Lack of or limited help
	Lack of recognition
	Social isolation
	Fatigue/burnout
	Concern about impacts on students
Stepping up	Workload uplift
	Innovating and reimagining
	Technical upskilling
Strategy	Creating new fieldwork models or partnerships
	Re-thinking or rewriting curriculum to make best use on-line technologies and platforms
	Creating new course content
Legacy impacts and Lessons	Greater academic and professional collaboration
	Technological upskilling
	Simulations
	Authenticity focus to education
	Some skills/competencies can't be taught on-line
	Online teaching requires a different approach and energy

### Disruption

Disruption (amid an environment of constant change) was the overarching theme in the educator narratives describing their experiences during the 2020–2021 period. This disruption was abrupt, forced and sustained, necessitating major changes to the way educators felt, practiced and adapted (professionally and personally). This disruption appears to have resulted in sustained changes to work and academic practice.

*The suddenness of change was an initial challenge. My recollection is that a university wide email was circulated late on a Monday afternoon and we had only days to act: move a work location to home, move entire curricula to online, think about access to equipment and other resources*.

*Moving classes online had to be done at short notice, literally overnight in one instance*.

Changes were significant and across multiple domains of educators' reality. This included changes to work practices, location, access to colleagues, workload, and work duration. These changes spilled over into educators' personal lives, as they were working from home and experiencing uncertainty and anxiety about how bad the situation would get, and how long they would be impacted.

*Change was a constant, could happen quickly and was not always well communicated. And that meant there was also uncertainty bubbling away. The speed and amount of change were cognitively and emotionally exhausting. We would put a plan in place, but a government and institutional policy would change and mean that plan was no longer workable so you would need to start again*.

*Working from home all day every day also blurred the lines between work and down-time, creating work life balance issues particularly during the height of the pandemic when our whole lives were at home*.

### Stress

Work related stress was a broadly felt impact of disruption and change, exacerbated by uncertainty, persistent change, the duration of the disruption and a lack of preparedness. This added to personal stress associated with the fear of infection, concern for family and uncertainty about employment and career disruption. Additionally, educators were confronted with the stress and mental health challenges experienced by their students.

*The impact was huge.... there was not only the need for transferring teaching online and encouraging students' engagement, but also the need for supporting students' wellbeing and mental health*.

*The pandemic has seen levels of both diagnosed and undiagnosed anxiety in students and generalized stress levels escalate dramatically. Anecdotally, student resilience and ability to cope with change and stressors of daily life seems to be at an all-time low and this has persisted. Students often had difficulty accessing university mental health services. This has all placed an additional strain on staff who are not necessarily trained to deal with the mental health issues arising*.

*[When] students could be allocated placement, there was a lot of stress and anxiety from students about surviving a shift in full PPE, not knowing how to meet the emotional needs of patients, seeing distressing circumstances and not feeling supported, access to buddy [practitioners], access to support staff due to restrictions on movements between wards … and not feeling welcome in a stressed environment*.

### Stepping up

Taking responsibility and developing solutions was a widely shared response to the forced change experienced by educators during this period, often with a personal cost in terms of workload, fatigue, and job satisfaction. This provided positives for some of the educators in terms of an initial sense of shared purpose and later satisfaction in seeing students advance through the course despite the disruption.

*At the first few months (around March–May 2020), it felt like an adventurous new experience where everyone was like “yes, we can do this” and “we are in this together”. We created new strategies to keep our students engaged and to stay connected with colleagues. However, after a while the sense of adventure was replaced by boredom and extra pressure on staff to first ensure the quality of the teaching, second to engage students in online delivery and third to provide additional support to students whose wellbeing was compromised due to the pandemic*.

Stepping up for many was a lonely experience, under-supported and under-recognized by the institution. This was particularly relevant to academic workloads, requiring an uplift and prioritization of workload to shifting teaching materials on-line, working long hours to meet short timeframes and with limited institutional support. This prioritization was at the expense of other academic activities such as research.

*I saw an incredible increase in my workload, well beyond our “paid” 7.5-hour days. I commenced work at 7.30 a.m. in the morning and finished between 7.30 p.m*.−*10 p.m. I did this so that I could manage the workload from converting material to online or a covid-19 safe method, but also try to keep up with my research (and its transition from face to face to online)*.

*You were (re)developing curricula, teaching it, then (re)developing the next class. I remember doing 15 hour days for months. There didn't seem to be a choice*.

*The impact … in 2020–2021 on my workload was significant. Although unreported, I was working for at least 10 hours daily–sometimes over the weekend to support students who were unwell/unable to attend clinical placement (e.g., there was a student who could not leave his apartment due to apartment-wide lockdown)*.

*My research workload significantly decreased. One project was abandoned completely because the Focus Groups were not permitted to go ahead face to face and …the final data was not able to be collected. Additionally, the conference we were presenting our findings at was canceled. ….A second project was pushed to the back burner as the authors struggled for time to write, and then the wait time on the manuscript submission took 10 months, so the data is really old not and most likely the article will not go ahead*.

Educators reported having to abruptly reimagine how they taught and rapidly upskill in utilization of on-line platforms and related pedagogy/andragogy. This was particularly felt with respect to skills and practice-based learning requiring access to labs and clinical environments (access to both were curtailed for extended periods during the COVID disruption). This linked to subthemes that reflected a lack of preparedness for such a disruption countered by initiative and a strong desire to ensure student outcomes and wellbeing.

*There was no extra support to move online–you learned as you went and shared what you learned with others. I remember logging a ticket for help from IT–it took 6 months for me to hear back. They were stretched and their remit and scope changed overnight as well*.

*I taught some units with a face-to-face audience and an online audience simultaneously, despite having no technological support, no extra work time and there being a vastly different pedagogy between the two approaches*.

*Usually when people learn to use new technology this take[s] place over several weeks or longer to learn one new system at a time. Staff [who] were not previously required to be overly tech savvy were now asked to learn multiple new systems at once and move all of their content online within days*.

As the COVID disruption progressed, educators reported that the initial mobilization and effort to respond to the forced change became replaced with fatigue and anxiety (burnout) associated with the duration of the disruption (few expected it to last so long) and the under-recognized additional workload. Concern about the quality of teaching and learning and the wellbeing of students were additional themes expressed in the context of stressors experienced by educators.

*It was around mid-2021 when a sense of boredom and burnout took over. Students were losing interest in online tutorials and began to miss “uni life”. Some staff, too, found it difficult to maintain work-life balance and connections with peers*.

*There was a lot of messaging about looking after ourselves but the reality of workloads didn't match that. Burnout felt inevitable*.

*Managing expectations can be hard enough at the best of times but it became even more difficult; students understandably found the uncertainty just as unsettling as we did*.

### Strategy

The initial and urgent strategic priority experienced by educators was adapting to on-line learning necessitated by university and government mandated lockdowns. This required educators to adapt to working from home and quickly convert from on-campus and in person modes of teaching to on-line. For many this required major curriculum re-writes, development of on-line content and learning how to utilize on-line technologies and platforms. In addition to workload uplift and prioritization by existing academic staff to convert quickly to on-line, sessional staff were mobilized to assist with the additional workload. Furthermore, maintaining student engagement in the online space was a constant challenge, particularly with less confident students.

*The energy required to learn (and teach) online tends to be underestimated and it was really hard to find a balance in the first 12 months or so when units were fully online and this was the only option. Even within a single class, it was more tricky to keep the energy up*.

*Online teaching and learning is not the same as face to face and it's not simply a matter of knowing how to use certain tools, or even what tools exist. There still seems to be an emphasis on tips and tricks with digital tools rather than online pedagogy. Any use of tools needs a strong educational philosophical foundation*.

One of the major challenges experienced by educators was the loss of clinical and laboratory learning and teaching environments associated with workplace and health service lockdowns. This required an abrupt rethinking of how to teach clinical skills using simulations and on-line placement learning exposures.

### Unexpected positives, lessons, and legacy impacts

Enhanced collaboration between academics and support staff was a commonly reported positive impact of COVID disruption and forced adaptation and innovation required educators to reimagine teaching and learning.

*The situation created a climate that was solution focused and collaborative amongst courses, with resources being shared*.

*The significant impact of the loss of clinical placement resulted in the creation of an online clinical placement unit…. [which] combined asynchronous (e.g., case management planning, completion of clinical reasoning forms, self-directed research) and synchronous masterclasses. Swinburne and [another university in Victoria] were the only two universities in Australia that offered simulated clinical placement in response to COVID. We managed to get this innovative online clinical placement simulation unit externally accredited*.

*We were presented with a chance to really interrogate what we were teaching, why and how. Although this arose from necessity and was hard to do at speed and in the face of frequent upheaval, there was a need to think and act differently*.

The COVID disruption resulted in a reported rapid upskilling of educators in on-line learning, technology use, and the use of simulated patient experiences. This was largely self-directed out of need rather than institutionally supported. These impacts appear to be sustained legacies of the period.

*There is no doubt that learning new technologies created a very steep learning curve for many staff. However, these skills have now been learned and continued to develop. This rapid learning has enabled technology to be integrated into work life on an ongoing basis. Our canvas pages are also a lot more highly developed now due to a greater reliance on them since the pandemic*.

*[I was] not confident initially, but [my skills] have improved enormously over the pandemic. I have far improved understanding of what tools are available, how the tools work, and where the tools can be used to support teaching, learning and student engagement*.

*With universities tending to increasingly favor blended learning approaches we now find ourselves somewhat ahead of the game as many of our units are already blended to a large degree*.

The experience of teaching online consolidated educators' conviction that some of the core competencies required for allied health practice cannot be taught and/or assessed on-line or removed from clinical environments.

*To develop core competencies health professional students need the hands on experience that you can only get on campus, interacting with staff, other students and utilizing discipline specific equipment, assessments and resources*.

*I think online can work for theory if the material is interesting, engaging and students can see the relevance to their professional practice. I think distance teaching does not develop the essential technical skills, necessary level of competence or student confidence*.

## Discussion

Health professionals have been encouraged to share stories among colleagues about their responses to the COVID-19 pandemic (25) and, understandably, much of the early literature was descriptive (17). This study adds to the research field and specifically our understanding of the experiences of health professional educators during the pandemic. There are some similarities between our findings and other studies within uni-disciplinary contexts (18–20). Those similarities include needing to adapt quickly to changing circumstances and the emotional impact of various stressors. Our research differs however in that it is based within an interdisciplinary context.

Some participants in our study voiced a sense of impending burnout, but all remained in their roles which is consistent with findings from a study of Romanian kindergarten, primary, and middle school teachers which found that only 13% of were “high burnout risk” (i.e., likely to detach from the job in general as well as from the teaching community) (26). A key finding of our study was that despite the sustained COVID-19 disruptions being a stressful experience for all, there were unexpected positives. During this period, staff generated and tested multiple strategies that were ultimately successful in progressing students through their courses in a timely manner. This is consistent with findings from a longitudinal study of 1,626 Canadian teachers who reported increased efficacy for classroom engagement and a sense of accomplishment (27).

Table 4 summarizes some of the educator strategies used by our participants during COVID disruption. Now that face-to-face teaching and fieldwork have resumed without restriction, educators have continued to use some of these strategies, and have re-considered the ways in which they can effectively teach professional competencies. Some of these strategies, particularly online and blended placements, can also have implications for healthcare workforce training in rural and regional areas. Salter et al. (28) discusses the unique opportunities that tele-practice and remote supervision brought for rural health placements following the pandemic. Some of these include technological upskilling, opportunities for additional training and support due to reduced travel time, and shifts in attitudes.

**Table 4:** Strategies generated and tested in COVID-disrupted years.

**Strategy**	**Outcome**
Simulated fieldwork experience (using actors, mannequins, digital avatars etc.)	All student fieldwork hours achieved (although some delayed).
Modified placements–online project-based placements completed during disruption period in-lieu of clinical placements (clinical placements completed the following semester)	Course progression maintained for all students in all nursing and allied health courses.
Hybrid hospital placements: online (e.g., students research a condition on-line then present to the supervisor) combined with in-person placement components	Positive feedback from our students and supervisors: •Online placement component decreased performance pressure (e.g., students could research a condition at any time, decreased risk of patient safety compromise) •Decreased health risk (i.e., COVID transmission) for the students, hospital staff and patients.
Sessional workforce mobilization	This reduced workload for permanent staff and allowed more effective online teaching (e.g., having two facilitators for online workshops or tutorials allowed small group work in breakout rooms).
Adoption of micro-credentials and skills-based modules	Integration of competencies/micro-credentialling into curriculum. For example, students had the opportunity to complete introductory online modules in telehealth and resilience and these modules have been embedded within the broader curricula of certain programs.
Creation of an online clinical placement unit combining asynchronous (e.g., case management planning, completion of clinical reasoning forms, self-directed research) and synchronous masterclasses.	Recognition by professional accrediting board–praised for innovative online clinical placement simulation.

One of the key legacies has been a move to more authentic blended learning. Moving theoretical content online has increased face to face class time available for problem based learning and practical or clinical skill acquisition. Online teaching methods have been shown to work well for content that is usually delivered in lectures or classroom sessions. One US study, which included 837 student evaluations from 191 public higher education institutions, found that the rapid transition to online instruction in 2020 did not negatively impact student performance and may have marginally increased these marks (29). It is worth noting, however, that no health professional degrees were included in the sample. The types of unexpected positives described by our participants are consistent with those found in a qualitative study of written reflections from a group of 115 international educators from medicine, nursing, dentistry, and the allied health professions (30). In that study, the authors used an “appreciative enquiry” method to focus on positive experiences of participants from one cohort of specific course, whereas our approach was deliberately generic to allow a range of experiences (negative, neutral, or positive). The “educational silver linings” were universal, including new teaching and assessment methods in classrooms and clinical settings, making new professional connections, and the rapid acquisition of new technological skills.

The impact of the pandemic on academic staff acted as a “double edge sword”; it brought several uncertainties yet opportunities to upskill capabilities, skills, confidence, and resilience; particularly in relation to use of technology and online teaching tools. Prior to the pandemic, Flavell et al. (31), explored experiences of science-based academic staff in relation to ongoing technological changes and the impact on teaching practices. They found that adapting to technological advances leads to professional development, increased level of confidence and opportunities to engage with unfamiliar tools that resulted in changing attitudes and beliefs toward use of technology. In our study, similarly, participants reflected that adapting to online teaching posed a challenge and “extra” work for them, but we might anticipate that the transition was more uniquely challenging due to the level of disruption associated with the pandemic. Nonetheless, adapting eventually led to learning skills, capability and confidence. This is an important lesson particularly as universities are moving toward blended and technology-informed learning that offers flexibility and engages students in decision-making. This is consistent with the experiences of other academics experiencing COVID disrupted teaching, who have described the emergence of student-centered “agile” responses to ensure course delivery (32, 33).

Another prominent finding from our study was the “opportunities in disguise” that the pandemic brought for curriculum review, evaluation and redesign. Several participants spoke about upskilling their capabilities, innovation, partnership and re-design of learning and teaching activities to ensure acquisition of the core competencies. Health professional education is historically slow to change (34, 35). The speed and scale of disruption caused by the pandemic has prompted Department level changes to academic practice including strengthening the governance role of discipline leads, reconsidering student engagement in the process of competency attainment and focusing on authenticity of learning exposures and assessment. This finding is in line with a 2020 study by Currie et al. (36) that explored the impact of the pandemic in medical radiation science teaching in an Australian University. The authors discussed that although the pandemic posed several challenges on higher education teaching, it also brought opportunities for evaluation and re-design of the teaching activities to enhance learning and ensuring sustainability and cultural safety.

We note, with interest, the largely absent mention by participants of relationships with students and colleagues and self-care strategies. That is not to say that participants did not care about their students or colleagues or their own health, but rather that relationships and self-care were not strongly identified in the participants' narratives. It might indicate that participants elected to focus on the “work” as a way of managing the emotional disruption of increasing work, shifting goals and frequent change. It might also indicate that some form of distance was a means of self-care. This absence may also be a result of the narrative prompts.

## Limitations

The participant sample was not large however it was drawn from a small interdisciplinary population. As a result, it may not be possible to transfer the findings to other settings, but this is not typically the objective of qualitative research, nor case-based research, which is inherently context-specific. This study deliberately did not report participants' demographic details as they could have potentially identified the participants.

The survey was administered after the height of the pandemic and this may have affected participants' recollections. However, this may also have resulted in more tempered recollections, with the passage of time allowing participants time to reflect and perspectives be less influenced by the direct emotional impact of the immediate experience.

## Conclusion

The abrupt closure of university campuses during the COVID-19 pandemic resulted in significant challenges resulting from the rapid transition to on-line or blended delivery in health professional education. There were profound impacts on educators and students alike with stressors related to increased workloads, learning new technologies whilst maintaining educational quality, supporting student engagement, teaching practical skills, and disruption to clinical placements and fieldwork. Despite this, unexpected opportunities were created, resulting in innovations in curriculum delivery, simulated and modified placements and acquisition of new technological skills including on-line platforms.

It is, however, important to ensure ongoing monitoring and critical evaluation of the impact of strategic learning and teaching responses to COVID disruption on competency attainment, as this is the primary measure of effective workforce preparation by universities. Whilst many universities have embraced blended learning and other remote learning platforms, this may come at the expense of student: academic interaction and incidental and unstructured learning opportunities. Participant narratives highlighted that, as a minimum, practical skill acquisition to reach the required level of competence in nursing and allied health professions remains the domain of face-to-face teaching.

## Data availability statement

The raw data supporting the conclusions of this article will be made available by the authors, without undue reservation.

## Ethics statement

The studies involving human participants were reviewed and approved by Swinburne University of Technology HREC. Written informed consent for participation was not required for this study in accordance with the national legislation and the institutional requirements.

## Author contributions

LO'B, RH, SB, LD, JT, and RL participated in conceptual discussion of key components of the methodology. LO'B and RH conducted the qualitative analysis. LO'B drafted the article and incorporated all feedback. LO'B, SB, JT, and ND responded to and actioned reviewer comments. All authors provided critical revisions of the article, contributed to the article, and approved the submitted version.
